# Cortical Neuroprotective Mechanisms of Exercise Training in Post-Traumatic Brain Injury: A Systematic Review

**DOI:** 10.3390/ijms27010052

**Published:** 2025-12-20

**Authors:** Farhan Yousaf, Sean Kao, Shahid Ishaq, Shin-Da Lee

**Affiliations:** 1 Department of Physical Therapy, Graduate Institute of Rehabilitation Science, China Medical University, Taichung 110122, Taiwan; farhany571@gmail.com; 2 Department of Biology, Johns Hopkins University, Baltimore, MD 21218, USA; skao11@jhu.edu; 3 PhD Program in Healthcare Science, College of Healthcare Science, China Medical University, Taichung 110122, Taiwan; shahidishaq53@gmail.com

**Keywords:** aerobic exercise, cerebral cortex, neurorehabilitation, TBI

## Abstract

Traumatic brain injury (TBI) causes cortical dysfunction by increasing oxidative stress, neuroinflammation, apoptosis, and mitochondrial dysregulation, and impairing neurotrophic signaling and neurogenesis. This systematic review aimed to evaluate the effectiveness of exercise training on cortical molecular dysregulation and motor function in post-TBI. Following PRISMA 2020 guidelines, PubMed, EMBASE, and Web of Science were searched up to August 2025. Of 1173 records, 35 studies involving exercise training in post-TBI animal models were included. Exercise training protocols included voluntary wheel running, treadmill running, and swimming, with durations ranging from 7 to 63 days. Study quality was assessed using the CAMARADES checklist. Exercise training increased cortical glutathione and Na^+^/K^+^-ATPase activity and reduced oxidative stress in post-TBI. It reduced microglial, astrocytic reactivity, and pro-inflammatory markers, including IL-1β and TNF-α expression in post-TBI. It also reduced caspase activity while increasing heat shock protein 20 (HSP20), thereby downregulating cortical apoptosis in post-TBI. It enhanced motor function, cortical neurogenesis, and neurotrophic factors signaling, including BDNF, in post-TBI. Exercise training improved motor function and cortical neuroprotection by reducing oxidative stress, neuroinflammation, and apoptosis, while enhancing neurotrophic signaling and neurogenesis in post-TBI rodents, but the regulation of let-7c, IL-6, and mitochondrial function remained unclear. (PROSPERO: CRD420251073725)

## 1. Introduction

Traumatic brain injury (TBI) causes progressive neurodegeneration, particularly in the cerebral cortex, and impairs motor function, with up to 53% of individuals reporting functional limitation after 12 months following mild TBI [1,2]. It also involves molecular dysregulation characterized by increased oxidative stress, neuroinflammation, apoptosis, mitochondrial dysfunction, and decreased neurotrophic signaling and neurogenesis, which impair cortical function [3,4,5,6,7]. Exercise training has emerged as an effective non-pharmacological intervention capable of modulating these molecular mechanisms and improving cortical function in post-TBI [7,8].

Oxidative stress arises from reduced antioxidant enzyme activity, leading to accumulation of reactive oxygen species (ROS) and lipid peroxidation products such as protein carbonyls, thiobarbituric acid reactive substances (TBARS), and malondialdehyde (MDA) [9]. These changes decrease sodium–potassium adenosine triphosphatase (Na^+^/K^+^-ATPase) activity [10] and are promoted by nicotinamide adenine dinucleotide phosphate (NADPH) oxidase activity [11]. Neuroinflammation involves pro-inflammatory markers and microglial and astrocytic reactivity, characterized by increased tumor necrosis factor-α (TNF-α), interleukin-1β (IL-1β), ionized calcium-binding adaptor molecule-1 (IBA1), glial fibrillary acidic protein (GFAP), and signal transducer and activator of transcription-3 (STAT3) expression [12,13]. It also involves dysregulation of nuclear factor kappa-light-chain-enhancer of activated B cells (NF-κB) activation and consequent interleukin-6 (IL-6) expression [14]. Apoptosis is characterized by caspase activation, cytochrome c release, and dysregulated microRNAs, such as microRNA-21 (miR-21), which alters the B-cell lymphoma 2 (Bcl-2)/Bcl-2-associated X protein (Bax) ratio [15]. Additional markers include BH3-interacting domain death agonist (Bid) and p53 upregulated modulator of apoptosis (PUMA), which facilitate apoptosis-inducing factor (AIF) translocation and DNA fragmentation [16]. Mitochondrial dysfunction is marked by reduced peroxisome proliferator-activated receptor gamma coactivator 1-alpha (PGC-1α) activity, leading to decreased adenosine triphosphate (ATP) synthesis and mitochondrial respiration [17].

Neurotrophic signaling is altered through decreased brain-derived neurotrophic factor (BDNF), neurotrophin-3 (NT-3), insulin-like growth factor 1 (IGF-1), tropomyosin receptor kinase B (TrkB), and cAMP response element-binding protein (CREB) expression [18]. CREB further regulates PGC-1α expression, linking decreased neurotrophic signaling to mitochondrial dysregulation [19]. Suppressed neurogenesis is indicated by reduced Ki67^+^ and neuronal nuclei (NeuN^+^) cells, along with dysregulated vascular endothelial growth factor A (VEGF-A) [20], erythropoietin (EPO), synapsin-1, and growth-associated protein 43 (GAP-43) expression, which collectively disrupt synaptic plasticity and cortical repair [21].

Exercise training modulates molecular dysregulation, as reported in previous reviews on the effects of exercise training on peripheral immune response [22], hippocampal neuroprotection [23], neurovascular dysregulation [24], cognitive recovery [25], and cerebrovascular dysregulation [26] in post-TBI. In parallel, converging evidence from other conditions, including Alzheimer’s disease [27], Parkinson’s disease [28,29], ischemic stroke [30], multiple sclerosis [31], spinal cord injury [32], epilepsy [33], and aging [34], demonstrates that exercise training exerts potential neuroprotective effects through modulation of neurobiological mechanisms [35]. Collectively, the subcortical structures involved in the pathophysiology of these neurological conditions are critical for motor execution. The cerebral cortex plays a fundamental role in motor function [36,37]. Cortical plasticity is key to functional recovery following injury, particularly in response to aerobic exercise training intervention [38]. Focusing on cortical mechanisms allows for a more direct interpretation of how exercise training influences molecular changes at the primary site of injury, which is important. However, evidence regarding the effectiveness of exercise training on motor function and cortex molecular mechanisms in post-TBI remains limited. Therefore, this systematic review aimed to summarize the effectiveness of exercise training on motor function and molecular dysregulation, including oxidative stress, neuroinflammation, apoptosis, mitochondrial function, neurotrophic signaling, and neurogenesis, in post-TBI.

## 2. Methods

This systematic review followed the PRISMA 2020 guidelines [39] and was registered on PROSPERO: CRD420251073725. A predefined PICO framework was employed to conduct this systematic review on preclinical TBI studies with traumatic brain injury (TBI) as the population, exercise training as the intervention, sedentary groups as the comparator, and cortical molecular regulation and motor function as the outcomes.

### 2.1. Search Strategy

A systematic search of PubMed, EMBASE, and Web of Science was conducted up to August 2025, using terms including “traumatic brain injury”, “exercise training”, and “cortex” (Supplementary File: S1). Additional studies were identified by screening the reference lists of included studies. The search library was maintained in EndNote V21.

### 2.2. Eligibility Criteria

After removing duplicates, title and abstract screening were performed, followed by full-text screening. Studies involving the post-TBI animal model, without any restrictions regarding age, species, or sex, that administered ≥7 days of exercise training compared to sedentary control, were included. Reviews, case reports, conference papers, and in vitro studies, and those focused on brain regions other than the cerebral cortex and employing interventions other than exercise training or lacking a sedentary control, were excluded.

### 2.3. Data Extraction

Data extraction encompassed study characteristics such as author, year of publication, TBI induction method, animal model details (sample size, species, age, sex, weight), and the exercise training protocol. Outcome measures included motor function and cortical molecular dysregulation, including oxidative stress, neuroinflammation, apoptosis, mitochondrial function, neurotrophic signaling, and neurogenesis assessed by real-time or quantitative PCR for gene expression, ELISA, Western blot, or immunofluorescence for protein expression and molecular changes. Data were extracted from figures, tables, graphs, and the text of included studies. Three independent reviewers systematically conducted the literature search, extracted data, and assessed risk of bias; disagreements were resolved by discussion with the senior author.

### 2.4. Risk of Bias

Risk of bias was assessed using the CAMARADES checklist [40] comprising ten items: (1) published in peer-reviewed journal, (2) temperature control, (3) randomization, (4) allocation concealment, (5) blinding assessment of outcome, (6) anesthesia avoidance with marked intrinsic properties, (7) post-TBI animal model, (8) sample size calculation, (9) compliance with regulatory requirements, and (10) conflict of interest statement. Each item was scored 1 for “yes” and 0 for “no” or “unclear”.

## 3. Results

**Figure 1:**
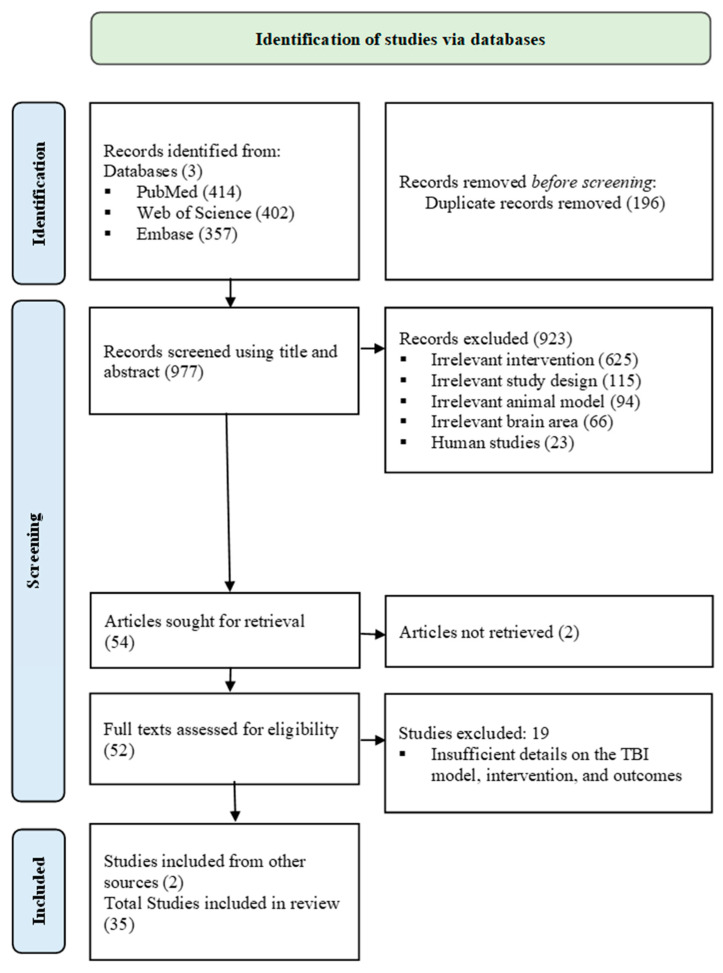
PRISMA 2020 flowchart.

A total of 1173 articles were found across PubMed (414), Web of Science (402), and EMBASE (357). After removing 196 duplicates, 977 articles’ titles and abstracts were screened; 923 were excluded due to irrelevant study design (115), human studies (23), non-TBI animal model (94), brain location other than cortex (66), or not meeting exercise training criteria (625). Following, 54 articles were sought for retrieval, and 2 were not found. Full-text (52) articles were screened, and 19 were excluded due to insufficient detail on the TBI induction method, exercise training protocol, or not measuring the targeted outcomes. Additionally, 2 articles were included from other sources, and a total of 35 studies were included in this systematic review (Figure 1).

**Figure 2:**
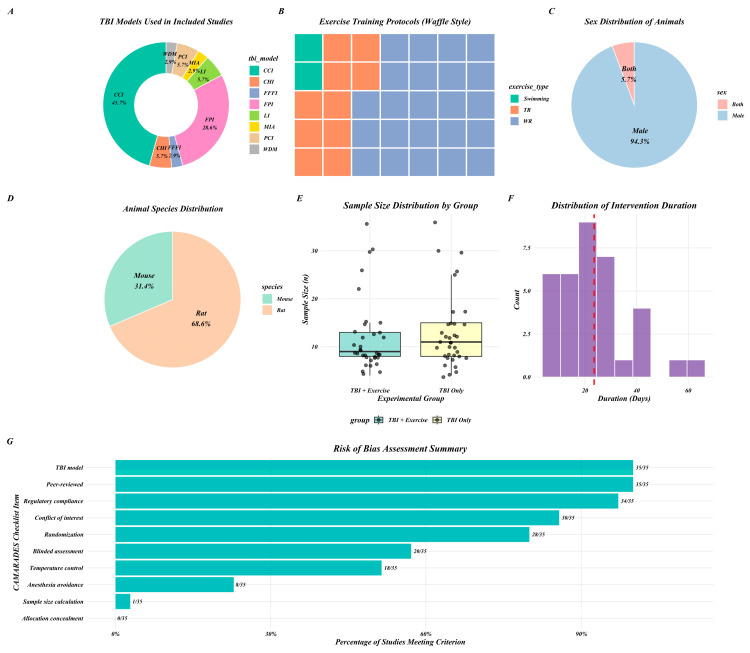
Graphical representation of key study characteristics (**A**–**F**) and RoB summary (**G**). CCI (controlled cortical impact); CHI (closed head injury); FPI (fluid percussion injury); PCI (pneumatic controlled injury); FFFI (free-fall weight impact injury); LI (lateral impact); WDM (weight-drop model); MIA (Marmarou’s impact acceleration). Red line in (**F**) shows mean exercise duration.

Risk of bias was assessed using the 10-item CAMARADES checklist (Table 1). The average score was 6.3/10, ranging from 4–8. All included studies were peer reviewed and involved a post-TBI animal model (Figure 2).

Across the included studies, 46% used the controlled cortical impact (CCI) TBI induction method, 29% used fluid percussion injury (FPI), and 25% used pneumatic controlled injury (PCI), free-fall weight impact (FFFI), lateral impact (LI), closed head injury (CHI), weight-drop model (WDM), and Marmarou’s impact acceleration (MIA). Exercise training included wheel running (WR) in 66% of studies, treadmill running (TR) in 23%, and swimming in 11%. In terms of animal species, 60% of studies used rats and 40% used mice. The majority of experiments were conducted exclusively in male animals, while two studies included both male and female animals. The average sample size was 12 animals in the TBI exercise training (TBI + Ex) group and 13 animals in the TBI group. Intervention durations varied between 7 and 63 days of exercise training, with an average of 21 days. All outcome measures represent the effectiveness of post-TBI treatment (TBI + Ex) compared to the sedentary group (Figure 2 and Table 2).

### 3.1. Oxidative Stress

Exercise training increased sodium–potassium adenosine triphosphatase (Na^+^/K^+^-ATPase) activity, glutathione/oxidized glutathione (GSH/GSSG) ratio, superoxide dismutase (SOD) activity, and let-7c microRNA (let-7c) expression, whereas it reduced protein carbonyl content (carbonyl), thiobarbituric acid reactive substances (TBARS), nicotinamide adenine dinucleotide phosphate oxidase (NADPH oxidase) activity, and malondialdehyde (MDA) levels as compared to the TBI group [6,48,58,60,62,64,65,66,70].

### 3.2. Inflammatory Pathway

Exercise training reduced expression of IL-1β, TNF-α, ionized calcium-binding adapter molecule 1 (IBA-1), glial fibrillary acidic protein (GFAP), signal transducer and activator of transcription 3 (STAT3), IL-18, inducible nitric oxide synthase (iNOS), cluster of differentiation 16 (CD16), chitinase-like protein 3 (Ym-1), arginase 1 (Arg-1), cluster of differentiation 206 (CD206), myeloperoxidase (MPO) activity, complement C1q subcomponent subunit B (C1qb), cluster of differentiation 68 (CD68), galectin-3, and NLR family pyrin domain-containing 3 (NLRP3), as well as allograft inflammatory factor 1 (Aif1), Il1b, Tnf, Il12, Ifng and C–C motif chemokine ligand 2 (Ccl2) mRNAs expressions, compared to the TBI group [5,6,14,43,44,48,50,52,53,56,62,65,67,71]. IL-6, IBA-1, GFAP, and integrin subunit alpha M (Itgam)-mRNA expressions were heterogeneous across studies, with reports of increases, decreases, or no change in the TBI + Ex group compared to the TBI group [6,42,43,47,62].

### 3.3. Apoptotic Pathway

Exercise training increased heat shock protein 20 (HSP-20), heat shock protein 70 (HSP-70), telomerase activity, telomere length, and heat shock protein family A member 1A (Hspa1a)-mRNA compared to the TBI group [14,45,61,71]. Pro-apoptotic regulators, including miR-21, miR-92a, miR-874, BH3-interacting domain death agonist (Bid), BCL2-binding component 3 (Bbc3) mRNAs, cytochrome c, apoptosis-inducing factor (AIF), caspase activation, DNA fragmentation, and single-stranded DNA (ssDNA), were reduced [5,7,60,71], whereas apoptosis-associated speck-like protein containing a CARD (ASC) and 4′,6-diamidino-2-phenylindole (DAPI) labelled cells remained unchanged in the TBI + Ex group as compared to the TBI group [43,47].

### 3.4. Mitochondrial Function

Exercise training increased PGC-1α expression, mitochondrial respiration, and ATP synthesis as compared to the TBI group in three studies only [49,61,70].

### 3.5. Neurotrophic Factors

Exercise training increased BDNF, NT-3, insulin-like growth factor 1 (IGF-1), Bdnf mRNA, and cAMP response element-binding protein (Creb)-mRNA expression [45,49,57,61,67,71], whereas one study reported no significant change in BDNF [59] and another observed reduced pCREB, CREB, protein kinase C (PKC), calcium/calmodulin-dependent protein kinase II (CaMKII), and mitogen-activated protein kinase II (MAPKII) expression, as compared to the TBI group [51].

### 3.6. Neurogenesis

Exercise training increased neuronal nuclei-positive (NeuN^+^) cells, neural stem cells, Ki-67, neurosphere formation, neuronal density, vascular endothelial growth factor A (VEGF-A), synapsin-I, growth-associated protein 43 (GAP-43), and Vegfa, erythropoietin (Epo), miR-138, miR-124, and DNA methyltransferase 1 (Dnmt1) mRNA expressions [5,14,42,44,52,54,55,60,61,63,68,71], whereas it reduced corticosterone-like activity, zinc finger protein 268 (Zif268), and 20S proteasome activity as compared to the TBI group [66]. In two studies, neuron loss was unchanged, and synapsin-I was decreased in the TBI + Ex group as compared to the TBI group [51,64].

### 3.7. Motor Function

Exercise training improved reaching activity, motor movement, wrist motor function, swing velocity, motor coordination, and recovery [5,6,7,41,46,50], whereas it reduced reaching abnormalities, neurological severity scores, veterinary coma scale scores, and cortical dysfunction, as compared to the TBI group [44,53,65,69]. Fine motor activity remained unchanged in only one study in the TBI + Ex group as compared to the TBI group [41].

**Figure 3:**
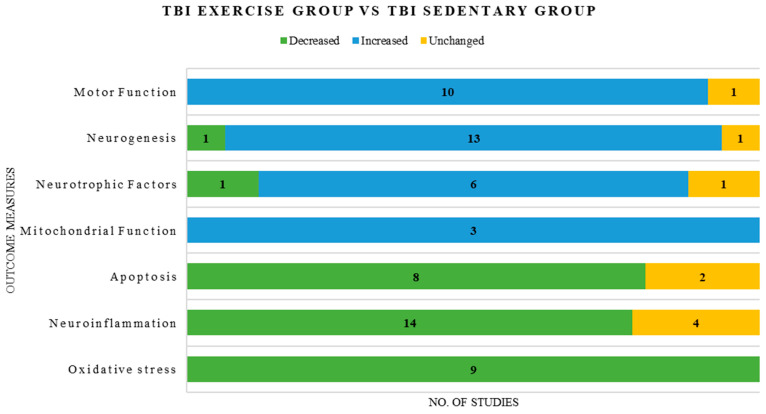
Graphical representation of consistent and inconsistent outcome measures reported in the included studies.

Overall, oxidative stress markers demonstrated consistency across most of the included studies compared with other molecular pathways, including neuroiflammation, apoptosis, neurotrophic signaling, neurogenesis, and motor function, which showed heterogeneous findings, with reports of increases, decreases, and no changes in outcome measures. (Figure 3)

## 4. Discussion

**Figure 4:**
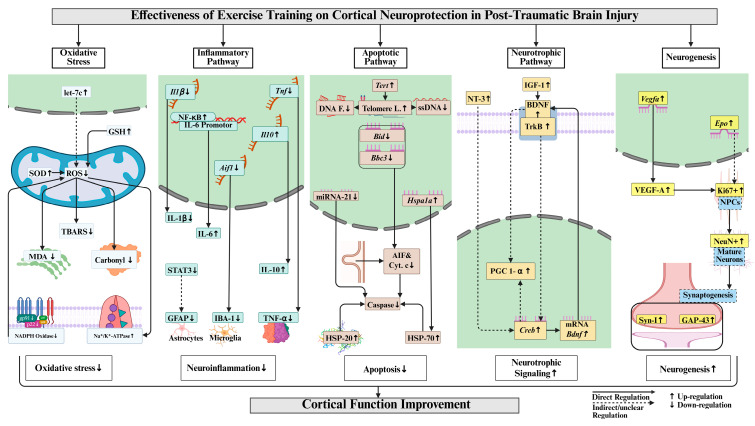
Key cortical neuroprotective mechanisms of exercise training in post-TBI. Abbreviations and their full forms are as follows: GSH (glutathione); SOD (superoxide dismutase); ROS (reactive oxygen species); TBARS (thiobarbituric acid reactive substances); MDA (malondialdehyde); NADPH (nicotinamide adenine dinucleotide phosphate); Il1β/IL-1β (interleukin-1 beta); Tnf/TNF-α (tumor necrosis factor-alpha); NF-κB (nuclear factor kappa-light-chain-enhancer of activated B cells, transcription factor); Il10/IL-10 (interleukin-10); STAT3 (signal transducer and activator of transcription 3); GFAP (glial fibrillary acidic protein); IBA-1 (ionized calcium-binding adapter molecule 1); Aif1 (allograft inflammatory factor 1); Tert (telomerase reverse transcriptase); DNA F. (DNA fragmentation/damage); Telomere L. (telomere length); ssDNA (single-stranded DNA); Nod (nucleotide-binding oligomerization domain); Bax (Bcl2-interacting killer); Bcl2 (B-cell lymphoma 2); AIF (apoptosis-inducing factor); Cyt. c (cytochrome c); HSP-70/HSP-20 (heat shock protein 70/20); BDNF (brain-derived neurotrophic factor); IGF-1 (insulin-like growth factor 1); TrkB (tropomyosin receptor kinase B); PGC-1α (peroxisome proliferator-activated receptor gamma coactivator 1-alpha); Creb (cAMP response element-binding protein); Vegfa/VEGF-A (vascular endothelial growth factor A); Ki67 (proliferation marker protein Ki-67); SPCs (stem/progenitor cells); NeuN (neuronal nuclei); Syn-1 (synapsin-1); and GAP-43 (growth associated protein 43). ↑ increase, ↓ decrease, ↔ no change.

This systematic review comprehensively demonstrates that exercise training regulates cortical function and molecular mechanisms in post-TBI rodents. Exercise training attenuated cortical oxidative stress by increasing antioxidant enzyme activity, including Na^+^/K^+^-ATPase and glutathione, thereby reducing lipid peroxidation and protein oxidation in post-TBI. Exercise training attenuated cortical neuroinflammation through the suppression of pro-inflammatory markers and by modulating microglial and astrocytic reactivity while also enhancing anti-inflammatory markers in post-TBI. Exercise training reduced cortical apoptosis in post-TBI by downregulating proapoptotic markers and caspase activation. Exercise training promoted cortical neurotrophic signaling post-TBI by increasing BDNF and IGF-1 expression and supporting neurogenesis (Figure 4). Evidence regarding mitochondrial function regulation following exercise training remains limited in post-TBI rodents and requires further investigation.

Oxidative stress was reduced by exercise training, restoring cortical redox balance and enhancing endogenous antioxidant defenses in post-TBI. The consistency in the upregulation of cortical Na^+^/K^+^-ATPase activity by exercise training highlights the restoration of neuronal ionic balance post-TBI [48,58,64]. The antioxidative role of *let-7c* after exercise training in post-TBI remains unclear due to limited evidence and requires further investigation [60]. Antioxidant signaling pathways, including the Nrf2 pathway, were not studied in any included studies, limiting translational interpretation. However, existing literature supports the antioxidative role of exercise in neurodegenerative diseases [72].

Neuroinflammation was attenuated by exercise training through the suppression of pro-inflammatory cytokines and modulation of microglial and astrocytic reactivity within the cerebral cortex in post-TBI. Variability in GFAP, IBA-1, and IL-6 expressions across studies likely reflects methodological differences in TBI severity and exercise training protocol, such as acute exercise training in severe TBI showing no attenuation in GFAP and IBA-1 expressions [6,42,43,47]. Two studies reported IL-6 changes consistent with anti-inflammatory signaling [14,62] while one study considered it pro-inflammatory [48] and one study did not mention its role clearly [6]. A systematic review of human studies also reported conflicting evidence, noting that serum, CSF, and parenchymal IL-6 elevations are associated with poor post-TBI outcomes [73]. Therefore, it is difficult to conclude that exercise training reduces inflammation specifically through IL-6 regulation, although reduction in other anti-inflammatory markers, including IL-1β and TNF-α, supports the potential of exercise training in reducing neuroinflammation. While evidence on the anti-inflammatory role of exercise training in neurodegenerative diseases supports these findings in post-TBI, preclinical studies that systematically examine inflammatory pathway regulation across varying TBI severities are still needed to inform clinical translation, particularly regarding exercise timing, dose, and intensity [74].

Apoptosis was mitigated by exercise training via downregulation of pro-apoptotic signaling mechanisms and reduction of DNA fragmentation in post-TBI. This protection against programmed cell death was largely consistent among included studies, supporting the neuroprotective effect in post-TBI [5,7,60,71]. One study reported no change in ASC, which needs further investigation to clarify the role of exercise training in modulating cortical inflammasome-mediated apoptosis in post-TBI [43]. Another study that used a severe TBI model also showed no improvement in apoptosis after short-term exercise training in post-TBI [47]. The observed anti-apoptotic effect of exercise training in post-TBI aligns with prior literature demonstrating that it suppresses neuronal apoptosis [75]. Based on available evidence, it is plausible that exercise training can attenuate neuronal apoptosis in TBI patients, modulating transcriptional regulators of apoptosis, provided that frequency, intensity, and time of exercise training are closely monitored with reference to the severity of the TBI.

Neurotrophic factor signaling was enhanced by exercise training, marked by upregulation of BDNF-TrkB signaling, supporting neuronal survival and cortical plasticity post-TBI. Variation in expressions of cortical BDNF, pCREB, CREB, PKC, CaMKII, and MAPKII in two studies may reflect differences in exercise training onset and time in post-TBI [51,59]. Despite this variation in included studies, the existing evidence in neurotrauma also supports this review’s finding that the BDNF-TrkB signaling pathway is one of the key mechanisms that promotes neuroprotection [76]. Future studies exploring cortical CREB, MAPK, and PKC regulation after exercise training in post-TBI are necessary to better understand neurotrophic signaling mechanisms.

Neurogenesis increased in the cerebral cortex with exercise training through molecular and structural changes, progressing from neural stem cell proliferation to increased expression of synaptic proteins, in post-TBI. Exercise training consistently enhanced cortical neuronal survival and synaptic protein expression while stimulating neural stem cell activity post-TBI in most of the included studies [54,55,63]. Some inconsistencies in synaptic protein expressions and neuron loss in only two different studies reflect that acute exercise training for a short period of time is not beneficial in moderate to severe TBI [51,64]. Our review’s findings on the effects of exercise training in cortical neurogenesis post-TBI are supported by existing evidence on neurodegenerative diseases [77,78]. The integration of these cortical molecular pathways culminates in a significant and consistent improvement in motor function following exercise training post-TBI. However, reports of unchanged performance in specific fine motor tasks highlight the sensitivity of functional outcomes to the site of injury [41].

The translation of these preclinical findings to clinical practice requires careful consideration of TBI severity and exercise training parameters, such as onset, intensity, total duration, and frequency per week. Some studies have reported an improvement in motor function outcomes and molecular regulation in the cortex after exercise training, suggesting a possible correlation between underlying neurobiological mechanisms and functional improvement [5,6,7,50]. Moderate TBI severity models CCI and FPI that can cause focal or diffuse injury demonstrated the most consistent benefits from exercise training, particularly with exercise training initiated after the acute phase and lasting for at least 3 weeks, while one study employing an acute short-term voluntary exercise training program reported worse molecular outcomes [51]. This aligns with clinical evidence suggesting optimal recovery when voluntary aerobic activity begins after the acute inflammatory phase and progresses gradually [79]. Exercise training lasting 21–63 days was more frequently associated with favorable molecular and motor outcomes, although beneficial effects were also observed in some shorter-duration protocols, suggesting a potential influence of intervention duration on recovery. However, substantial heterogeneity in exercise training protocols and TBI severity limits definitive conclusions and highlights the need for further investigation on how exercise training duration affects the recovery process and underlying mechanisms in different severities of TBI. The predominance of male subjects in the included studies (94%) represents a significant limitation for clinical translation, as human TBI affects both sexes, with previous literature showing differences in pathophysiology and recovery [80]. The site of injury is crucial when devising an exercise training program, as it may improve neurobiological markers in other areas of the brain but not at the site of injury when applied short term [41]. Thus, it is important to understand graded long-term exercise training effects in preclinical and clinical experiments to study molecular changes and clinical outcomes, respectively.

Although all included studies used rodent TBI models, preclinical research has substantially improved itsbtranslational relevance through standardized injury induction models, functional outcome assessments, and biomarker integration [81,82]. While differences in anatomy, immune responses, and injury biomechanics remain, parallel evaluation of biomarkers measurable in both animals and humans strengthens cross-species interpretation [83,84]. For instance, in patients with post-traumatic disorders of consciousness, fluid biomarkers, including BDNF and microRNAs, reflect ongoing neurodegeneration, neuroinflammation, and plasticity mechanisms that are also observed in experimental rodent models of TBI [85]. Furthermore, neurobiological mechanisms are implicated in other neurodegenerative conditions, including Alzheimer’s disease and aging, where exercise-induced myokine signaling and BDNF regulation promote neuroprotection, suggesting shared mechanisms involved in healthy aging and neuroprotection [34,86,87]. Non-pharmacological therapeutic measures are supported by clinical evidence showing that they can modulate inflammatory markers while inducing neuroprotective mechanisms, supporting the translational perspective of this review [88,89]. Collectively, the current literature supports the translational potential of animal model TBI experimental research and highlights clinically relevant neuroprotective mechanisms that bridge experimental and human studies.

### Limitations and Future Implications

This systematic review evaluated the effectiveness of exercise training in cortical neuroprotection in post-TBI; however, several limitations must be acknowledged. Most of the included studies used predominantly male animals and had relatively small sample sizes, limiting the generalizability of the findings. Moreover, evidence regarding some cortical molecular mechanisms of exercise training in post-TBI, such as the role of anti-inflammatory IL-6, mitochondrial function, and *let-7c* signaling, remains limited or inconclusive across the included studies. Evidence is also limited because of the exclusion of non-English studies. TBI models used in the included studies do not represent all severities of TBI clinical populations, such as skull fracture, military blast TBI, or severe TBI in road traffic accidents. The findings of this review do not establish a causal relationship between outcome measures but rather suggest a possible association between molecular mechanisms and motor function. Future studies should incorporate larger samples, both sexes, longitudinal molecular assessments, varying TBI models, such as skull fracture or blast TBI, and evaluate understudied molecular pathways across varied exercise training protocols before clinical translation of these findings.

## 5. Conclusions

Exercise training improved motor function and modulated molecular mechanisms, including oxidative stress, neuroinflammation, apoptosis, neurotrophic signaling, and neurogenesis in the cortex, supporting its therapeutic potential for cortical neuroprotection in post-TBI rodents. However, several pathways, notably mitochondrial regulation, IL-6 signaling, and *let-7c*, require further exploration; translation to clinical practice should consider injury severity, lesion location, and carefully defined exercise frequency, onset, intensity, and duration.

## Figures and Tables

**Table 1:** CAMARADES Risk of Bias Assessment: (1) published in peer-reviewed journal, (2) temperature control, (3) randomization, (4) allocation concealment, (5) blinding assessment of outcome, (6) anesthesia avoidance with marked intrinsic properties, (7) post-TBI animal model, (8) sample size calculation, (9) compliance with regulatory requirements, and (10) conflict of interest statement.

Study	1	2	3	4	5	6	7	8	9	10	Total Score
Adkins 2015 [41]	1	0	1	0	0	0	1	0	1	1	5
Amorós-Aguilar 2020 [42]	1	1	1	0	1	0	1	0	1	1	7
Barrett 2025 [43]	1	1	1	0	1	1	1	0	1	1	8
Chen 2013 [44]	1	1	1	0	1	1	1	0	1	1	8
Chio 2017 [14]	1	1	1	0	1	0	1	0	1	1	7
Chou 2018 [45]	1	0	1	0	0	0	1	0	1	1	5
Combs 2016 [46]	1	0	1	0	1	1	1	0	1	1	7
Crane 2012 [47]	1	0	1	0	0	0	1	0	1	1	5
de Castro 2017 [48]	1	1	1	0	1	0	1	0	1	1	7
Ferguson 2020 [49]	1	0	1	0	1	0	1	1	1	1	7
Gan 2022 [50]	1	0	1	0	1	0	1	0	1	1	6
Griesbach 2004 [51]	1	1	0	0	0	0	1	0	1	0	4
Gu 2014 [52]	1	1	0	0	1	0	1	0	1	0	5
Hu 2023 [53]	1	1	1	0	1	1	1	0	1	1	8
Itoh 2011a [54]	1	1	1	0	0	0	1	0	1	0	5
Itoh 2011b [5]	1	1	1	0	0	0	1	0	1	0	5
Jacotte-Simancas 2015 [55]	1	1	1	0	1	0	1	0	1	1	7
Karelina 2021 [56]	1	0	1	0	1	0	1	0	1	1	6
Koo 2013 [57]	1	0	1	0	0	0	1	0	1	1	5
Lima 2009 [58]	1	1	0	0	0	0	1	0	1	1	5
Martínez-Drudis 2021 [59]	1	1	1	0	1	0	1	0	1	1	7
Miao 2015 [60]	1	1	0	0	1	0	1	0	1	0	5
Mota 2012 [6]	1	1	0	0	0	0	1	0	1	0	4
Mychasiuk 2016 [61]	1	1	1	0	1	1	1	0	1	1	8
Piao 2013 [62]	1	1	1	0	1	0	1	0	1	0	6
Rafie 2024 [7]	1	1	0	0	1	0	1	0	1	1	6
Sánchez-Martín 2024 [63]	1	1	1	0	1	1	1	0	1	1	8
Silva 2013 [64]	1	1	1	0	1	0	1	0	1	1	7
Soltani 2020 [65]	1	1	1	0	1	0	1	0	1	1	7
Szabo 2010 [66]	1	1	1	0	0	0	1	0	1	0	5
Tabor 2019 [67]	1	1	1	0	1	0	1	0	1	1	7
Taylor 2015 [68]	1	0	1	0	0	0	1	0	1	1	5
Wang 2024 [69]	1	1	1	0	1	1	1	0	1	1	8
White 2023 [70]	1	0	1	0	1	0	1	0	1	1	6
Zhao 2015 [71]	1	1	1	0	0	1	1	0	1	1	7

**Table 2:** Summary of study characteristics and outcome measures in the traumatic brain injury (TBI) exercise training group compared to the TBI sedentary group. CCI (controlled cortical impact); CHI (closed head injury); FPI (fluid percussion injury); PCI (pneumatic controlled injury); FFFI (free-fall weight impact injury); LI (lateral impact); WDM (weight-drop model); MIA (Marmarou’s impact acceleration); n (TBI exercise training group sample vs TBI group sample); A (age in weeks); S/Sp (sex/species); Wt (weight); WR (wheel running); TR (treadmill running); t (duration of each exercise session, minutes or hours per day); T (total duration of intervention, days); f (frequency of exercise sessions, days per week); v (velocity of treadmill running, meters per minute); Na^+^/K^+^ ATPase (sodium–potassium adenosine triphosphatase); TBARS (thiobarbituric acid reactive substances); gp91^phox (catalytic subunit of NADPH oxidase); p22^phox (stabilizing subunit of NADPH oxidase); MDA (malondialdehyde); GSH (reduced glutathione); GSSG (oxidized glutathione); SOD (superoxide dismutase); IBA1/Aif1 (ionized calcium-binding adapter molecule 1); Cybb (cytochrome b-245 beta chain); Tnf (tumor necrosis factor-α); Itgam (integrin alpha-M/CD11b); GFAP (glial fibrillary acidic protein); Il1b (interleukin-1β); STAT3 (signal transducer and activator of transcription-3); IL-6 (interleukin-6); NF-κB (nuclear factor kappa-B); Il12 (interleukin-12); Ifng (interferon-γ); Ccl2 (chemokine ligand-2); Il10 (interleukin-10); Tgfb (transforming growth factor-β); NLRP3 (NOD-like recep-tor family pyrin domain-containing-3); IL-18 (interleukin-18); CD68 (cluster of differentiation-68); iNOS (inducible nitric oxide synthase); CD16 (cluster of differ-entiation-16); Ym1 (chitinase-like protein-1); Arg1 (arginase-1); CD206 (cluster of differentiation-206); MPO (myeloperoxidase); C1qB (complement component 1q subunit-B); Galectin-3 (galectin-3); ASC (apoptosis-associated speck-like protein containing a CARD); HSP70/Hspa1a (heat shock protein-70); HSP20 (heat shock protein-20); ssDNA (single-stranded DNA); miR-21 (microRNA-21); miR-92a (microRNA-92a); miR-874 (microRNA-874); Tert (telomerase reverse transcriptase); TL (telomere length); Bid (BH3-interacting domain death agonist); Bbc3/Puma (Bcl-2 binding component-3); Caspase (caspase); Cyt-c (cytochrome-c); AIF (apoptosis-inducing factor); PGC-1α (peroxisome proliferator-activated receptor-γ coactivator-1α); ATP (adenosine triphosphate); BDNF (brain-derived neurotrophic factor); TrkB (tropomyosin receptor kinase-B); CREB/Creb1 (cAMP response-element binding protein); p-CREB (phosphorylated CREB); PKC (protein kinase-C); CaMKII (calcium/calmodulin-dependent protein kinase-II); p-MAPKII/MAPKII (mitogen-activated protein kinase-II); NT-3 (neurotrophin-3); IGF-1 (insulin-like growth factor-1); NeuN (neuronal nuclei); Syn-I (synapsin-I); GAP43 (growth-associated protein-43); NSC (neural stem cells); Ki-67 (cell proliferation marker); miR-138 (microRNA-138); miR-124 (microRNA-124); Dnmt1 (DNA methyltransferase-1); Zif268/Egr1 (early growth response-1); VEGF-A (vascular endothelial growth factor-A); EPO (erythropoietin); NSS/mNSS (neurological severity score/modified neurological severity score); VCS (veterinary coma scale); DAPI (4′,6-diamidino-2-phenylindole); LC3-II (microtubule-associated protein light chain-3-II); Maoa (monoamine oxidase-A); Nr3c1 (nuclear receptor subfamily-3 group-C member-1); Hmox1 (heme oxygenase-1).↑ increase, ↓ decrease, ↔ no change.

Study	Study Characteristics		Outcome Measures						
Author-Year	TBI Model	Exercise Training Protocol	Oxidative Stress	Inflammatory Pathway	Apoptosis	Mitochondrial Function	Neurotrophic Factors	Neurogenesis	Motor Outcomes
Adkins 2015 [41]	TBI: CCI n: 9 vs. 9 A: 12 wks S/Sp: Male rats Wt: 250–350 g	WR t: 6 h/day T: 28 d	-	-	-	-	-	-	Reaching activity ↑, fine motor activity ↔
Amorós-Aguilar 2020 [42]	TBI: CCI n: 13 vs. 17 A: 7 wks S/Sp: Male rats Wt: 230 g	WR T: 21 d	-	IBA1 ↔	-	-	-	NeuN+ ↑	
Barrett 2025 [43]	TBI: CCI n: 8 vs. 12 A: 10 wks S/Sp: Male mice	WR T: 56 d		mRNA Tnf ↓, Itgam ↔, Gfap ↔, and Il1b ↓. GFAP ↔, STAT3 ↓, IBA1 ↓	ASC↔	-	-	-	-
Chen 2013 [44]	TBI: CHI n: 6–12 vs. 6–12 A: 7 wks S/Sp: mice	TR t: 1 h/day T: 14 d v: 9–13.5 m/min	-	IBA1 ↓	-	-	-	NeuN+ ↑	NSS ↓
Chio 2017 [14]	TBI: FPI n: 8/8 S/Sp: Male rats Wt: 310 g	TR T: 21 d f: 5 d/wk	-	IL-6 ↑, NF-κB binding at IL-6 Promotor ↑	HSP70 ↑, Apoptosis ↓	-	-	Syn-I ↑	-
Chou 2018 [45]	TBI: FPI n: 10 vs. 10 S/Sp: Male rats Wt: 265 g	WR T: 24 d t: 30–60 min, v: 20–30 m/min	-	-	HSP 20 ↑	-	BDNF ↑, TrkB ↑	-	-
Combs 2016 [46]	TBI: CCI n: 22 vs. 25 A: 14 wks S/Sp: Male rats	WR t: 6 h/day + reach T: 28 d	-	-	-	-	-	-	Reach accuracy ↑, motor movement ↑, wrist motor function ↑
Crane 2012 [47]	TBI: PCI n: 6 vs. 8 A: 7 wks S/Sp: Male rats Wt: 290 g	WR T: 19 d	-	GFAP ↔, IBA ↔	DAPI ↔	-	-	-	-
de Castro 2017 [48]	TBI: FPI n: 6 vs. 6 S/Sp: Male rats Wt: 250–350 g	Swimming t: 60 min/d T: 40 d f: 5 d/wk	Na^+^/K^+^-ATPase activity ↑	TNF-α ↓, IL-6 ↓, MPO activity ↓	-	-	-	-	-
Ferguson 2020 [49]	TBI: CHI n: 7 vs. 7 A: 5 wks S/Sp: Male rats Wt: 100–140 g	WR T: 18 d	-	-	-	PGC-1α ↑	BDNF ↑	-	-
Gan 2022 [50]	TBI: FPI n: 13 vs. 12 A: 5 wks S/Sp: Male mice	WR T: 36 d f: 6 d/wk	-	GFAP ↓	-	-	-	-	Swing velocity ↑
Griesbach 2004 [51]	TBI: FPI n: 4 vs. 4 S/Sp: Male rats Wt: 250–300 g	WR T: 7 d	-	-	-	-	p-CREB ↓, CREB ↓, PKC ↓, CAMKII ↓, p-MAPKI ↓, MAPKII ↓	T and p-Syn I ↓,	-
Gu 2014 [52]	TBI: CCI n: 13 vs. 13 A: 16 wks S/Sp: Male mice	WR T: 21 d	-	GFAP ↓	-	-	-	NeuN+ ↑, GAP43 ↑	-
Hu 2023 [53]	TBI: FFFI n: 12 vs. 15 A: 24 wks S/Sp: Male mice	WR T: 7 d	-	mRNA Il1b ↓, Il12 ↓, Ifng ↓, Ccl2 ↓, Il10 ↑, Tgfb ↑. NLRP3 ↓, IL-1β ↓, IL-18 ↓, IBA1 ↓, CD68 ↓, iNOS ↓, CD16 ↓, Ym-1 ↓, Arg-1 ↓, CD206 ↓	-	-	-	-	NSS ↓
Itoh 2011a [54]	TBI: CCI n: 36 vs. 36 A: 10 wks S/Sp: Male rats Wt: 200–250 g	TR t: 30 min/d T: 7 d v: 22 m/min	-	-	-	-	-	NSC ↑, Ki-67 ↑, neurospheres ↑	-
Itoh 2011b [5]	TBI: PCI n: 26 vs. 26 A: 10 wks S/Sp: Male rats Wt: 200–250 g	TR t: 30 min/d T: 7 d v: 22 m/min	-	GFAP ↓	ssDNA ↓	-	-	NeuN+ ↑	cerebral function ↑
Jacotte-Simancas 2015 [55]	TBI: CCI n: 9 vs. 11 A: 7 wks S/Sp: Male rats Wt: 250 g	WR T: 20 d	-	-	-	-	-	NeuN+ ↑	-
Karelina 2021 [56]	TBI: CCI n: 15 vs. 15 A: 4–6 wks S/Sp: Male mice	TR t:10–30 min/d T: 13 d v: 6–15 m/min	-	IBA1 ↓	-	-	-	-	-
Koo 2013 [57]	TBI: CCI n: 10 vs. 10 S/Sp: Male rats Wt: 250–300 g	WR T: 21 d t: 15 min/d	-	-	-	-	NT-3 ↑	-	-
Lima 2009 [58]	TBI: FPI n: 8 vs. 8 A: 13 wks S/Sp: Male rats Wt: 220–320 g	Swimming T: 30 d t: 60 min/d f: 5 d/wk	Carbonyl ↓, TBARS ↓, Na^+^/K^+^-ATPase ↑, and its α1 subunit activity ↑	-	-	-	-	-	-
Martínez-Drudis 2021 [59]	TBI: CCI n: 9 vs. 10 A: 7 wks S/Sp: Male rats	WR T: 25 d	-	-	-	-	BDNF ↔	-	-
Miao 2015 [60]	TBI: CCI n: 30 vs. 30 A: 16 wks S/Sp: Male mice	WR T: 21 d	let-7c ↑	-	miR-21 ↓, miR92a ↓, miR-874 ↓	-	-	miR-138 ↑, miR124 ↑	-
Mota 2012 [6]	TBI: FPI n: 8–9 vs. 8–9 A: 13 wks S/Sp: Male rats Wt: 220–260 g	TR T: 28 d	Na^+^/K^+^-ATPase activity ↑	IL-1β ↓, TNF-α ↓, IL-6 ↔, IL-10 ↑, MPO activity ↓	-	-	-	-	Motor function ↑
Mychasiuk 2016 [61]	TBI: LI n: 8 vs. 11 S/Sp: Male/Female rats	WR T: 7 d	-	-	mRNA Tert ↑, TL ↑		BDNF ↑, Pgc1-α ↑, Igf-1 ↑	mRNA Dnmt1 ↑	-
Piao 2013 [62]	TBI: CCI n: 15 vs. 15 A: 10 wks S/Sp: Male mice	WR T: 63 d	gp91^phox and p22^phox ↓	IL-1β ↓, IL-6 ↑, IL-10 ↑, C1qB ↓, Gelactin-3 ↓	-	-	-	-	-
Rafie 2024 [7]	TBI: WDM n: 8 vs. 8 S/Sp: Male rats Wt: 250–300 g	TR t: 30 min/d T: 40 d f: 5 d/wk	-	-	Apoptosis ↓	-	-	-	Motor coordination & function ↑
Sánchez-Martín 2024 [63]	TBI: CCI n: 9 vs. 12 A: 6 wks S/Sp: Male rats Wt: 250 g	WR T: 9 d	-	-	-	-	-	NeuN+ ↑	-
Silva 2013 [64]	TBI: FPI n: 9 vs. 11 S/Sp: Male rats Wt: 250–300 g	TR; T: 20 d f: 4–5 d/wk	GSH ↑, GSH/GSSG ↑, carbonyl ↓, TBARS ↓, SOD ↑, Na/K ATPase ↑	-	-	-	-	Neuron loss ↔	-
Soltani 2020 [65]	TBI: MIA n: 12 S/Sp: Male rats Wt: 180–210 g	TR t: 30 min/d T: 40 d f: 5 d/wk v: 20–25 m/min	MDA ↓, Carbonyl ↓	IL-1β ↓	-	-	-	-	VCS ↓
Szabo 2010 [66]	TBI: FPI n: 5 vs. 4 S/Sp: Male rats Wt: 250–300 g	WR T: 14 d	Carbonyl ↓	-	-	-	-	Syn-I ↑, Corticosterone-like Activity ↓, Zif268 ↓, 20S proteasome ↓	-
Tabor 2019 [67]	TBI: LI n: 8 vs. 15 A: 3 wks S/Sp: Male rats	WR T: 14 d	-	mRNA Aif1 ↓	-	-	mRNA Bdnf ↑	-	-
Taylor 2015 [68]	TBI: CCI n: 30 vs. 30 A: 20 wks S/Sp: Male mice	WR T: 42 d	-	-	-	-	-	mRNA Vegfa ↑, Epo ↑. VEGF-A ↑ EPO ↑	-
Wang 2024 [69]	TBI: FPI n: 8 vs. 8 S/Sp: Male rats Wt: 250–350 g	WR t: 30 min/d T: 21 d	-	-	-	-	-	-	mNSS ↓,
White 2023 [70]	TBI: CCI n: 5–7 vs. 5–7 A: 5 wks S/Sp: Male/Female mice	TR t: 10–30 min/d T: 13 d v: 6 m/min	GSH/GSSG ↑	-	-	Mito. Respiration ↑, ATP ↑	-	-	-
Zhao 2015 [71]	TBI: CCI n: 15 vs. 17 S/Sp: Male mice A: 10 wks	WR T: 28 d	-	IBA-1 ↓	mRNA Bid ↓ and Bbc3 ↓. Caspase activation ↓, Cyt c & AIF translocation ↓, mRNA Hspa1a ↑		mRNA Bdnf ↑, mRNA Creb1 ↑	Neuronal Density ↑	-

## Data Availability

No new data were created or analyzed in this study. Data sharing is not applicable to this article.

## Supplementary Materials

The following supporting information can be downloaded at: https://www.mdpi.com/article/10.3390/ijms27010052/s1.

## PROSPERO Registration No

CRD420251073725 https://www.crd.york.ac.uk/PROSPERO/view/CRD420251073725 accessed on 15 December 2025.
