# Supplementary material for: Cortical Neuroprotective Mechanisms of Exercise Training in Post-Traumatic Brain Injury: A Systematic Review
Source: Int J Mol Sci. 2025 Dec 20;27(1):52. doi: 10.3390/ijms27010052 (PMC12785403; doi:10.3390/ijms27010052)
Supplement: Supplementary file 1 [file ijms-27-00052-s001.zip › ijms-4045726-supplementary.pdf]

Search Strategy:

(1) “traumatic brain injury” OR TBI OR concussion OR head trauma OR closed head injury OR mild traumatic brain injury OR experimental brain injury; AND (2) “exercise” OR “exercise therapy” OR “physical fitness” OR “motor activity” OR physical activity OR aerobic exercise OR resistance training OR endurance training OR strength training OR treadmill training OR voluntary exercise OR forced exercise OR exercise intervention OR physical training; AND (3) “cerebral cortex” OR cortex OR cortical OR cortical volume OR cortical thickness OR cortical function OR cortical activity OR cortical connectivity OR cortical neuroplasticity OR prefrontal cortex OR motor cortex OR somatosensory cortex OR visual cortex OR cortical reorganization OR cortical response OR cortical atrophy OR motor function OR neuroplasticity OR synaptic plasticity OR long-term potentiation OR BDNF OR brain-derived neurotrophic factor OR neurogenesis OR mitochondria OR mitochondrial OR mitochondrial biogenesis OR oxidative phosphorylation OR ATP production OR oxidative stress OR mitochondrial respiration OR PGC-1α OR NRF1 OR cytochrome c oxidase.
